# The Role of Trace Metals in the Development and Progression of Prostate Cancer

**DOI:** 10.3390/ijms251910725

**Published:** 2024-10-05

**Authors:** Unathi Albertinah Tshoni, Thokozani P. Mbonane, Phoka C. Rathebe

**Affiliations:** Department of Environmental Health, Faculty of Health Sciences, Doornfontein Campus, University of Johannesburg, P.O. Box 524, Johannesburg 2006, South Africa; tshoniunathi@gmail.com (U.A.T.); tmbonane@uj.ac.za (T.P.M.)

**Keywords:** prostate, cancer risk, trace metals, epigenetics, environment

## Abstract

Over the years, prostate cancer (PCa) research has been of great interest, and trace metals have attracted a lot of attention due to their association with prostate cancer development and progression. PCa has a complex etiology, with genetic, environmental, and lifestyle factors being implicated. Trace metals such as zinc (Zn), mercury (Hg), selenium (Se), lead (Pb), cadmium (Cd), manganese (Mn), arsenic (As), and nickel (Ni) have garnered much attention in recent years, suspected of having direct links to the modulation of cancer risk and progression through their impacts on prostate cancer omics (genomics, epigenetics, proteomics, and transcriptomics). This has led to them being the subject of extensive research in this regard. In this review, we explored the influence of trace metals and offered a comprehensive analysis of the current knowledge on how trace metals affect the biology of prostate cancer at a molecular level by integrating findings from the recent literature to help suggest possible directions for future research.

## 1. Introduction

Prostate cancer (PCa) is one of the most prevalent malignancies in men, and its incidence is increasing globally. It represents a noteworthy health burden [1], and its development and progression are intricately influenced by genetic predisposition and environmental factors [2,3]. Among the environmental factors, trace metals have attracted a great deal of attention, as the literature has linked them to an increased risk of prostate cancer [4,5]. It is a malignancy known for its significant ancestral disparity [6] and considerable heritability [2], especially in men of African ancestry [7,8]. There is also literary evidence that variations in PCa incidence are influenced by geography and racial distribution, suggesting varying genetic susceptibility levels in populations [9,10]. In general, countries with a high Human Development Index (HDI) tend to have a higher incidence rate of cancer compared to countries with a low HDI [11].

Understanding the significance of trace metals in prostate cancer is vital for clarifying the processes by which the environment influences the development and progression of PCa [5]. Deciphering the complex relationship between genetic predisposition and environmental exposure has the potential to improve the comprehension of the onset and progression of cancer [12]. Even individuals without a documented history of trace metal exposure may still be susceptible to the epigenetic effects of these environmental contaminants, which can lead to the dysregulation of critical cellular processes and contribute to the development of prostate cancer [13,14]. Currently, prostate-specific antigen (PSA) is the practical screening method in Africa, which is known to have high mortality rates, and it has proven to be unreliable as a biomarker [15,16,17].

In South Africa, PCa is the most prevalent cancer in men [18], and the incidence rate has increased from 29 in every 100,000 men in 2007 [19] to 68 in every 100,000 men in 2018 [20]. In addition to being the most diagnosed cancer type, as of 2014, prostate cancer had a high mortality rate, accounting for 13% of male mortality in the country, and had the highest mortality for men globally [11,21]. Black South African men are disproportionately affected because of their racial group; it is more likely to be hereditary than in any other racial group [22]. Owing to these statistics, one of the long-term goals for the South African government in the National Development Plan 2030 is to “substantially decrease the incidence of noncommunicable diseases” [23]; as a result, much research has been focused on prostate cancer among South African men.

Aside from epidemiological risk factors, the most promising avenues of enlightenment regarding the etiology of prostate cancer reside in elucidating the biological mechanisms whereby distinguishing factors such as the environment, geographic location, diet, family history, and ancestry have influenced the development of prostate cancer. Being black, of older age, and having a family history of prostate cancer are well-established risk factors for prostate cancer. Citing environmental, genetic, lifestyle, and epigenetic risk factors has been a growing interest in recent years. This review examines the roles of cadmium, nickel, zinc, manganese, selenium, arsenic, lead, chromium, and mercury in prostate cancer, focusing on their effects on epigenetic modifications, genomic stability, transcriptomic profiles, and proteomic alterations. In this review, we evaluated the epigenetics, genomics, transcriptomics, and proteomics literature to identify the causative link between trace metal exposure and epigenetic, genetic, transcriptomic, and proteomic changes associated with prostate cancer.

## 2. Methodology

This review was conducted following Preferred Reporting Items for Systematic Reviews and Meta-Analyses (PRISMA) guidelines [24]. The search strategy was conducted between January and July 2024 using the databases of PubMed, Science Direct, Springer, Nature, Research Gate, Scopus, and Google Scholar with no language restrictions. The search terms employed were prostate cancer OR prostatic carcinoma OR prostatic AND trace metals OR zinc OR magnesium OR selenium OR cadmium OR lead OR mercury OR arsenic OR nickel [25]. The results were narrowed into articles published from 2014 to July 2024. After removing duplicates, the titles, abstracts, and keywords of the retrieved articles were screened for eligibility. Only studies conducted on human populations were included. In this review, the PECO (Population, Exposure, Comparator, and Outcome of interest) framework was used to facilitate the literature search. P accounted for patients whose samples revealed focus trace metals, and E was exposure to trace metals. The comparator (C) was the control group included in each study, and O accounted for the development and progression of prostate cancer.

The study selection criteria for the systematic review examining the role of trace metals in prostate cancer were clearly defined. The inclusion criteria consisted of observational studies conducted in humans aged 19 years or above with prostate cancer or benign tissue confirmation, focusing on one or a combination of trace metals, including lead, arsenic, cadmium, manganese, mercury, nickel, selenium, or zinc. Trace metal measurement methods such as serum, whole blood, urine, nails, hair, and tissue samples were required. Studies involving occupational or environmental exposure were also included. Studies needed to be published in peer-reviewed journals in English from 2014, and cohort, cross-sectional, and case-control studies were considered. Exclusion criteria encompassed studies with inadequate data or measurements on trace metals, studies of other diseases, reviews, preprints, conference papers, opinion papers, case reports, and editorials. Data extraction was performed using a Microsoft Excel spreadsheet. The information that was extracted from each study included the following: authors, year of publication, type of study, population, age (mean), samples used, methods used to measure, trace metals measured, and country of origin. All studies were screened for eligibility.

This review employed the Newcastle–Ottawa Scale (NOS) for cohort and cross-sectional studies [26] to evaluate the risk of bias (Table 1). Within the scope of this review, a total of 20 studies were examined thoroughly, revealing that 1 of these studies was categorized as having a moderate score, suggesting an increased likelihood of risk of bias pertaining to the measurement of outcomes, primarily due to a lack of external control and information on characteristics important for comparability and outcomes. Furthermore, 3 studies were evaluated as presenting moderate-to-strong scores (score = 6), suggesting a minimized risk associated with confounding variables. Most studies exhibited a low risk of bias (score ≥ 7), thus improving the confidence of the studies and minimizing information bias. This methodological rigor functions to reduce the potential for overestimating the relationship between trace metals and the risk of prostate cancer.

Nonetheless, it is essential to recognize that certain confounding factors, particularly dietary influences, were not adequately addressed; thus, the observed correlation between trace metal exposure and the incidence of prostate cancer necessitates scrutiny. The possible ramifications of unmeasured confounders may further constrain the reliability of the effect estimates derived from these studies. Overall, the quality of evidence linking trace metals to the progression of prostate cancer is regarded as low to moderate. This evaluation underscores the necessity for judicious interpretation and the pursuit of longitudinal studies within this critical domain. Table 2 describes the characteristics of the studies included in this review.

## 3. Results

The search on databases resulted in 203 studies that were relatively related to the search after removing duplicates. Once studies published before 2014 were removed, 90 studies were assessed by screening the title and abstract, and 11 were excluded as they focused on other cancers or diseases. The remaining 79 were assessed in full text, resulting in the exclusion of 1 preprint, 26 reviews, 4 animals/in vitro studies, and 25 that did not have results of interest. Of the remaining 23, 3 were excluded because we could not find the full text. The remaining 20 studies met the inclusion criteria and are subject to this review. A total of 16 studies are case controls [5,26,27,29,30,31,32,33,34,35,36,39,40,41,42,43] with varying trace metals; 2 are cross-sectional [28,37], and another 2 are cohort studies [38,44].

Five studies were conducted in Nigeria [26,27,28,34,42]; the United States [5,35], Saudi Arabia [29,33], and Canada [40,41] had two studies each. One of the cohorts had a group from Serbia and Croatia [38]. Sudan [32], Singapore [36], Taiwan [43], Pakistan [39], Turkey [31], Russia [30], Spain [37], and Poland [44] had one study each. Most of these studies used serum levels to measure the concentration of trace metals [5,26,27,29,31,32,34,35,36,37,38,39,42,43,44], followed by urine [5,27,35,41], toenails/nail [28,39,40,41], scalp hair [33,39]. The least used samples were tissue [5] and prostatic fluid [30]. The most used tool/machine to measure the concentrations of trace metals was ICP-MS [5,27,29,33,36,37,38,40,41,43,44], followed by AAS [26,31,32,34,39,42]. Hood et al. [40] also used HPLC along with ICP-MS. The least used tools were PIXE [28], EDXRF [30], and ICP-DRC-MS [35].

The key findings of these studies (Table 3) reveal that zinc deficiency or low concentrations of serum zinc are associated with the prevalence of PCa [26,27,29,30,32,33,34]. However, in keratinized material, PCa is associated with higher concentrations of zinc [28,39,40]. Selenium goes hand in hand with zinc; low concentrations in serum selenium are associated with PCa prevalence [29,31,33,34,42]. High cadmium concentrations are found in PCa patients [5,35,38,39], even if patients were exposed to chronic low levels of cadmium [27]. The serum levels of Mn were found to be in low concentrations in PCa patients [29] and were contradicted by the findings of Zaichick and Zaichick [31], recording higher levels of manganese. They were higher in hair and nails [33,39]. Lim et al. [36] and Tyagi et al. [5] showed a positive correlation between high As levels and PCa, but the Serbian group in the cohort recorded low levels of As in PCa patients [38]. This cohort also recorded high levels of Ni in the Croatian group and low levels in the Serbian group, and [37,39] also recorded High levels of Ni. The Croatian group [38] recorded high levels of Hg, and the Serbian group recorded low levels of Pb. However, [34,39] recorded high levels of Pb in cancer patients.

## 4. Discussion

The findings of this review showed that serum levels of zinc and selenium are significantly lower in PCa patients compared to controls, while Zn levels in hair and nails are higher in patients than in controls. Studies have shown that selenium and zinc both offer protection against prostate cancer. Zinc has been shown by Zaichick and Zaichick [30] to be essential for preserving prostate health because it prevents the growth of malignant cells. According to Wu et al. [35], selenium functions as a potent antioxidant that lowers oxidative stress and may therefore lessen the risk of cancer. Optimal levels of the essential trace element selenium are required by the prostate to carry out its functions, especially in the synthesis of melanoproteins that have an important function in preserving cellular DNA. In prostate cancer, there is a consistently low level of selenium in the prostate. This low level of selenium is due to a decrease in the activity of the selenoprotein [46,47,48].

Remarkably, a considerable decline in zinc concentration in the malignant gland versus normal tissue is observed in PCa. Others have also noted that zinc in prostatic intraepithelial neoplasia (preinvasive mode of PCa) was still detected in the tissue, but its concentration was lower than that in normal tissue [29,49,50]. The decline of zinc homeostasis appears to be a critical early event in the acquisition of malignancy during PCa initiation and progression. This change leads to the upregulation of the sodium-coupled transporter ASCT2, resulting in a switch from mitochondrial metabolism to glycolysis in prostatic cancer cells [49,51,52].

As reported by [26,27], cadmium has a significant carcinogenic potential. It has been demonstrated that long-term cadmium exposure increases oxidative stress, inflammation, and genotoxicity, all of which are major factors in the development of prostate cancer. Given that cadmium can mimic estrogenic activity and alter hormonal balance and signaling pathways, its involvement in prostate cancer is further complicated. It is commonly known that cadmium has carcinogenic qualities. Cadmium exposure has been reported to cause oxidative stress, DNA damage, and the disruption of cellular processes, all of which considerably increase the risk of prostate cancer [27,33]. The concentration of serum Cd is abated by smoking [5]. Cadmium is a transition metal and byproduct of zinc carbonate (ZnCO_3_) purification, widely distributed in the environment [53,54]. It is classified as a Group 1 carcinogen. Cigarette smoke and, especially, second-hand smoke are still the largest sources of exposure in the nonsmoking population today. Moreover, people exposed to cadmium through their work, such as those in the metal and battery industries, are also at risk [55]. Findings from population-based studies showed increased risks for prostate cancer associated with edible or airborne Cd exposure [38,56,57]. An epigenome-wide association study indicated that Cd exposure was linked to microRNA (miR) dysregulation [45].

According to Amadi and Aleme [26], lead is yet another toxic metal associated with prostate cancer. Lead exposure over an extended period can cause inflammatory reactions, which can aid in the growth of tumors. Prostate cancer is also linked to lead, an established environmental contaminant. According to research by Saleh et al. [29] and Amadi and Aleme [26], exposure to lead may cause oxidative stress and DNA damage, which could accelerate the development of prostate cancer. Lead’s carcinogenic effects are exacerbated by its disruption of cellular processes and interference with calcium signaling. Lead is a ubiquitous contaminant from the combustion of leaded gasoline and has generated special concern since it is dangerous to human health due to its toxicity and bioaccumulation [58,59]. However, there are limited reports on the carcinogenic effects of lead exposure, but it is known to disrupt normal DNA transcription [60].

Mercury is renowned for its neurotoxicity; however, Nsonwu-Anyanwu et al.’s [34] study highlights that mercury may also have a role in prostate cancer. Exposure to mercury may cause immunological dysregulation, oxidative stress, and chronic inflammation, all of which may contribute to the development of prostate cancer. Significantly associated with several malignancies, including prostate cancer, arsenic is a known carcinogen. Arsenic exposure causes oxidative stress, angiogenesis, and tumor formation [33]. It also alters cellular signaling pathways. Prostate cancer incidence has been found to be higher in areas with contaminated water sources when there is chronic exposure to arsenic. Mercury is a muck soil contaminant with its abuse as a fungicide and its industrial effluents, and mercury’s oncogenic potential in target organs is poorly understood [61]. Arsenic has been suspected due to elevated levels of this metal in drinking water [62].

Studies have demonstrated a correlation between nickel exposure and an elevated risk of prostate cancer. Nickel could cause cancer by altering gene expression, inducing epigenetic alterations, and disrupting cellular function. Of particular concern is its capacity to produce reactive oxygen species (ROS) and disrupt DNA repair pathways; [28,33] noted high concentrations of Ni in PCa patients, and the concentration correlated with the duration of exposure as well as age [37]. Nickel, which can enter the body via inhalation, digestion, implantation, or directly via the bloodstream, can be divided into two subtypes: water-soluble nickel (Ni II) and water-insoluble nickel (Ni IV). The two subtypes are metabolized within the body to form a reactive oxygen species that binds to DNA and causes damage [54,63,64]. The role of manganese is more complex; Tyagi et al. [5] suggest that although manganese is necessary for cellular processes, too much of it might cause oxidative stress and inflammation, which may raise the risk of cancer, while Lim et al. [36] reckon abnormalities in manganese levels may impair antioxidant defenses, which further links manganese to the development of prostate cancer. The duality of manganese emphasizes how crucial it is to keep the body’s levels at proper levels.

The studies [37,38] highlight the extensive influence of exposure to metals on the development of prostate cancer. Cancer can result from chronic exposure to toxic metals, which can occur via food sources, environmental contamination, or occupational dangers that compromise cellular homeostasis. These studies demonstrate how repeated metal exposures can have a cumulative effect that increases the risk of cancer. Qayyum and Shah [39] and Hood et al. [40] offer valuable perspectives on the ways in which metals trigger inflammation and oxidative stress, two major factors that contribute to prostate cancer. These findings highlight the necessity of thorough risk evaluations and preventative actions to lessen the health concerns associated with metal exposure.

Epidemiological evidence relates metal exposure to prostate cancer incidence. These studies highlight the significance of monitoring and controlling occupational and environmental exposures to lower cancer risk by reporting increased rates of prostate cancer in communities with substantial exposure to industrial metals [41,42]. Some of these studies recommend early identification and intervention measures, especially for populations that are at risk of exposure to hazardous metals from the environment or their jobs. These findings emphasize the necessity of focused public health campaigns to increase knowledge of metal toxicity and encourage exposure reduction techniques [43,44].

The exploration of trace metals’ impact on prostate cancer provides a comprehensive insight into their influence on disease risk. Cadmium, lead, nickel, mercury, and arsenic are strongly linked to an elevated prostate cancer risk. These metals contribute to cancer development through oxidative stress, DNA damage, inflammation, and disruption of cellular functions. Zinc and selenium are recognized for their potential protective roles. These essential trace elements help reduce oxidative stress and may decrease prostate cancer risk by supporting DNA repair and cellular health. The research emphasizes the importance of reducing exposure to harmful metals, especially in high-risk groups, while ensuring adequate intake of protective elements. This balance is crucial for lowering prostate cancer risk.

**Figure 1:**
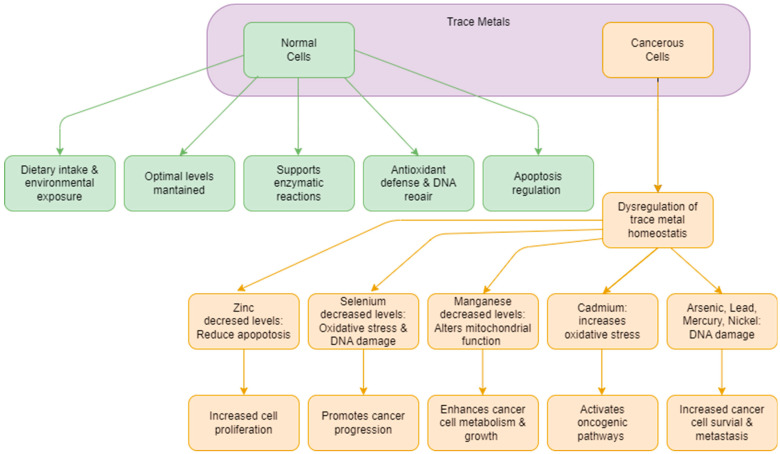
Schematic overview of risk pathways showing the effects of trace metals on normal and cancerous cells.

These studies also provide epidemiological evidence linking higher prostate cancer rates with exposure to toxic metals, particularly in industrial or contaminated environments. This underscores the need for public health interventions to reduce such exposures. However, further research is needed to better comprehend how these metals influence prostate cancer development and to develop effective prevention and intervention strategies. Public health policies should focus on reducing harmful exposures and promoting the dietary intake of protective trace elements like zinc and selenium. Overall, the central point is that while certain trace metals can significantly increase prostate cancer risk, others may offer protective benefits, highlighting the importance of managing environmental exposures and maintaining a balanced diet. Figure 1 delineates the multitude of intricate challenges and elements that must be considered inherent in establishing causality for each trace metal under scrutiny.

The exploration of the intricate relationships between trace metals and the etiology of cancer is paramount, as these elements possess a duality of effects that can either confer protective benefits or exacerbate malignancy, contingent upon exposure levels, biological environment, and individual genetic predisposition. Zinc serves as a critical cofactor in myriad cellular functions, encompassing DNA synthesis, repair, and immune homeostasis [47]. Investigations have posited that zinc deficiency may heighten oxidative stress, thereby increasing cancer vulnerability, while contrasting studies reveal that excessive zinc concentrations may catalyze tumorigenesis [48]. The paradoxical nature of zinc complicates its definitive role in oncogenesis. Its impact appears contingent upon the dose, exposure timing, and specific cancer type. For instance, zinc may operate as an antioxidant at physiological levels, yet excessive concentrations could instigate tumor progression, thus obscuring clear causal pathways [48,49]. Renowned for its antioxidant capabilities, selenium plays a pivotal role in the functionality of selenoproteins, which safeguard against oxidative damage [50]. Some research indicates that selenium supplementation may diminish cancer risk, particularly in prostate malignancies, while other studies report negligible or adverse effects at elevated dosages [51,52]. The dose–response relationship for selenium exhibits a non-linear pattern, suggesting that both deficiency and surplus can yield deleterious consequences. This complexity is further compounded by genetic variability in selenium metabolism, rendering universal conclusions elusive [53,54].

Manganese functions as a vital cofactor for several enzymatic processes integral to antioxidant defense and cellular metabolism [55,56]. Existing literature presents limited insights into manganese’s specific role in carcinogenesis, with some studies suggesting that abnormal manganese concentrations may influence cancer progression through modulation of oxidative stress and mitochondrial function [57,58]. Cadmium is an established carcinogen closely associated with oxidative stress, DNA damage, and the disruption of repair mechanisms [59,60]. There exists substantial evidence linking cadmium exposure to an increased incidence of lung, prostate, and renal cancers, particularly in occupational contexts [61,62,63]. While cadmium’s carcinogenic properties are well-documented, establishing a direct causal relationship is complicated by factors such as exposure duration, concurrent exposure to additional carcinogens, and individual genetic susceptibility [64,65,66]. Nickel is classified as a carcinogen primarily through its capacity to inflict DNA damage and modify gene expression [67]. The mechanisms by which nickel induces cancer involve epigenetic alterations and oxidative stress; however, the intricacies of these pathways remain incompletely elucidated. Additionally, concurrent exposures in industrial environments further complicate the isolation of nickel’s specific contributions [66,68].

Mercury induces oxidative stress and disrupts DNA repair processes. The carcinogenic potential of mercury may be contingent upon its form (e.g., organic versus inorganic), exposure levels, and duration. The variability in exposure sources and challenges in accurately quantifying long-term exposure further complicate the establishment of causality [60,69]. Lead, a notorious toxic metal, is primarily linked to neurological impairment but may also play a role in cancer development through mechanisms involving oxidative stress and the inhibition of DNA repair [60,69]. Epidemiological studies have yielded limited evidence suggesting a correlation between lead exposure and heightened risks of various cancers. The intricacies of establishing a causal relationship are exacerbated by confounding variables, including co-exposure to other carcinogens, lifestyle factors such as smoking, and fluctuations in exposure levels [70,71,72]. Arsenic is recognized as a potent carcinogen that impacts diverse biological pathways, including oxidative stress, inflammation, and the regulatory mechanisms of gene expression [73,74]. Chronic exposure to arsenic has been robustly linked to the incidence of skin, lung, bladder, and kidney cancers, particularly in regions with elevated arsenic levels in drinking water [75,76]. Although the evidence supporting arsenic’s carcinogenicity is compelling, individual susceptibility, genetic variability, and concurrent exposures can modulate cancer risk. Furthermore, the latency period between exposure and cancer manifestation complicates the direct linkage of early-life exposures to subsequent cancer development [12,73,77].

## 5. Strengths and Limitations

Various study designs used in the included studies, as well as different methodologies, sample sizes, populations, and locations, have led to variation in evidence and potentially contrasting conclusions. This might have impacted the outcomes by having inconsistent confounders. Also, case-control and cross-sectional studies may not provide a dynamic disease progression over time, suggesting a need for longitudinal studies when investigating the role of trace elements in prostate cancer development and progression. Different measurement methods, together with tools and samples, might have suggested inconsistent data conclusions.

Due to the scarcity of longitudinal studies within the literature, our review included studies of different designs to eliminate the risk of study selection bias. We also stretched the search year to include articles published 10 years ago and performed a risk of bias evaluation per study. However, due to the likelihood of change in trace metal exposure over time, there is a need for longitudinal epidemiological studies to evaluate exposure together with changes in lifestyle, different concentrations based on regulations, and other molecular factors.

## 6. Conclusions

The findings of these studies indicate that trace metals have a significant impact on the risk of developing prostate cancer, serving as both potential hazards and protective agents. Harmful metals such as cadmium, lead, nickel, mercury, and arsenic are consistently associated with an increased risk of prostate cancer due to their ability to cause oxidative stress, DNA damage, and inflammation. On the other hand, essential trace elements like zinc and selenium may provide protective effects by helping to reduce oxidative damage and support cellular health.

These results emphasize the importance of a balanced approach to managing exposure to trace metals. Public health efforts should prioritize reducing exposure to harmful metals, especially in high-risk environments, while also promoting adequate intake of protective metals such as zinc and selenium. This approach is essential for decreasing the overall impact of prostate cancer. Additionally, these studies underscore the significance of further research into the complex interactions between trace metals and prostate cancer. Understanding the specific mechanisms through which these metals influence the development of cancer will be crucial for developing targeted strategies for prevention and intervention. This research has important implications for public health, indicating that regulatory policies should prioritize both reducing exposure to harmful metals and promoting nutritional balance to lower the risk of prostate cancer.

## Figures and Tables

**Table 1:** Quality assessment of the studies included using Newcastle–Ottawa Scale (NOS).

	Studies	Selection				Comparability		Outcomes			Total
		Representative of the Exposed	Selection of External Control	Ascertainment of Exposure	Outcome of Interest Not Present at the Start	Main Factor	Additional Factor	Assessment of Outcomes	Sufficient Follow-Up Time (Cohort)	Adequacy of Follow-Up (Cohort)	
									Blind Assessment (Case and Cross Sectional)	Statistical Test (Case and Cross Sectional)	
1	Amadi and Aleme, 2019 [27]	*	*	0	*	*	*	*	*	*	8/9
2	Bede-Ojimadu et al., 2023 [28]	*	*	*	*	*	*	*	*	*	9/9
3	Igbokwe et al., 2021 [29]	*	*	*	*	*	*	*	*	*	9/9
4	Saleh et al., 2020 [30]	*	*	0	*	*	*	*	*	*	8/9
5	Zaichick and Zaichick, 2019 [31]	*	*	0	*	*	*	*	*	*	8/9
6	Eken et al., 2016 [32]	*	*	*	*	*	*	*	*	*	9/9
7	Abdelmajid et al., 2022 [33]	*	*	*	*	0	*	*	*	*	8/9
8	Saleh et al., 2017 [34]	*	*	*	*	*	*	*	*	*	9/9
9	Nsonwu-Anyanwu et al., 2022 [35]	*	*	*	*	*	*	*	*	*	9/9
10	Wu et al., 2021[36]	*	0	*	*	*	*	*	0	0	6/9
11	Tyagi et al., 2023 [5]	*	0	0	*	0	0	*	*	*	5/9
12	Lim et al., 2019 [37]	*	*	*	*	*	*	*	*	*	9/9
13	Alegre-Martínez et al., 2022 [38]	*	*	*	*	*	*	*	*	*	9/9
14	Pizent et al., 2022 [39]	*	*	*	*	0	*	*	0	0	6/9
15	Qayyum and Shah, 2014 [40]	*	*	*	*	*	*	*	*	*	9/9
16	Hood et al., 2023 [41]	*	*	*	*	*	*	*	*	*	9/9
17	Keltie et al., 2022 [42]	*	*	*	*	*	*	*	*	*	9/9
18	Onyema-iloh et al., 2014 [43]	*	*	0	*	0	0	*	*	*	6/9
19	Chang et al., 2018 [44]	*	*	*	*	*	*	*	*	*	9/9
20	Pietrzak et al., 2024 [45]	*	0	*	*	*	*	*	*	*	8/9

* Indicate visual quality assessment for each component in included studies.

**Table 2:** Overview and characteristics of studies used in this review.

Study	Author(s), Year	Study Type	Population (Mean Age)	Control Population	Sample Type(s)	Measurement Method/Tool	Focus Trace Metal(s)	Location
1	Amadi and Aleme, 2019 [27]	Case-control	440 (69.35)	220	Serum	Atomic Absorption Spectrometer (AAS)	Zn	Nigeria
2	Bede-Ojimadu et al., 2023 [28]	Case-control	273 (70.50)	99	Serum and Urine	Inductively Coupled Plasma Mass Spectrometry (ICP-MS)	Cd, Zn	Nigeria
3	Igbokwe et al., 2021 [29]	Cross-sectional	82 (71.72)	41	Toenails	Particle-Induced X-ray Emission (PIXE)	Zn	Nigeria
4	Saleh et al., 2020 [30]	Case-control	92 (67.17)	30	Serum	ICP-MS	Se, Zn, Mn	Saudi Arabia
5	Zaichick and Zaichick, 2019 [31]	Case-control	146 (61.32)	38	Prostatic fluid	Energy Dispersive X-Ray Fluorescent (EDXRF)	Zn	Russia
6	Eken et al., 2016 [32]	Case-control	131 (61.27)	40	Serum	AAS with a Zeaman Background Correction	Zn, Mn, Se	Turkey
7	Abdelmajid et al., 2022 [33]	Case-control	60 (N/A)	30	Serum	AAS	Zn	Sudan
8	Saleh et al., 2017 [34]	Case-control	174 (69.1)	52	Scalp hair	ICP-MS	Se, Zn, Mn	Saudi Arabia
9	Nsonwu-Anyanwu et al., 2022 [35]	Case-control	90 (66.60)	30	Serum	AAS	Se, Pb, Zn	Nigeria
10	Wu et al., 2021[36]	Cohort	5477 (66)	N/A	Blood, urine	Coupled Plasma Dynamic Reaction Cell Mass Spectrometry (ICP-DRC-MS)	Pb, Hg, As, Cd	United States
11	Tyagi et al., 2023 [5]	Case-control	256 (N/A)	N/A	Tissue, urine and serum	ICP-MS	As, Cd, Ni, Pb	United States
12	Lim et al., 2019 [37]	Case-control	255 (N/A)	114	Serum	ICP-MS	Mn, Zn, As, Se, Cd, Pb	Singapore
13	Alegre-Martínez et al., 2022 [38]	Cross-sectional	92 (72.2)	46	Serum	ICP-MS	Ni	Spain
14	Pizent et al., 2022 [39]	Cohorts	194 (N/A)	91	Whole blood and serum	ICP-MS	As, Cd, Cr, Hg, Ni, Pb	Croatia and Serbia
15	Qayyum and Shah, 2014 [40]	Case-control	140 (56.77)-B 134(56.01)-SH 120(55.18)-N	66 67 60	Blood, Scalp Hair, Nails	AAS	Cd, Mn, Ni, Pb, Zn	Pakistan
16	Hood et al., 2023 [41]	Case-control	88(60.90)	44	Toenails	ICP-MS and with High-Performance Liquid Chromatography (HPLC)	As, Mn, Ni, Zn, Se, Cd, Hg, Pb,	Canada
17	Keltie et al., 2022 [42]	Case-control	576(61.70)-TN 152 (61)-U	400 114	Toenails Urine	ICP-MS	As, Mn, Ni, Zn, Se, Cd, Hg, Pb,	Canada
18	Onyema-iloh et al., 2014 [43]	Case-control	100 (N/A)	50	Serum	AAS	Zn, Se	Nigeria
19	Chang et al., 2018 [44]	Case-control	60 (73.40)	23	Serum	ICP-MS	Cd, Ni, Hg, Pb, Zn, As	Taiwan
20	Pietrzak et al., 2024 [45]	Cohort	338 (N/A)	N/A	Serun	ICP-MS	Zn, Se	Poland

**Table 3:** Notable and key findings from the studies in this review.

Author(s), Year	Key Findings
Amadi and Aleme, 2019 [27]	Prostate cancer patients are characterized by zinc deficiency.
Bede-Ojimadu et al., 2023 [28]	Chronic exposure to low levels of cadmium may be associated with a heightened risk of PCa in individuals with insufficient zinc levels. Those with low zinc status may be more vulnerable to cadmium-related PCa. Urinary cadmium levels did not show a significant disparity between PCa patients and controls. These research findings should be considered in the development of public health initiatives aimed at reducing cadmium exposure and enhancing zinc intake through dietary measures, particularly given the prevalent zinc deficiency in Nigeria.
Igbokwe et al., 2021 [29]	This investigation revealed a higher concentration of zinc in the toenails of men with prostate cancer compared to men of similar age without the disease. However, no correlation was found between zinc levels in the toenails and PSA levels or Gleason scores.
Saleh et al., 2020 [30]	Reduced levels of selenium, zinc, and manganese may play a crucial role in initiating prostate cancer.
Zaichick and Zaichick, 2019 [31]	The study showed that Zn levels were much lower in the prostatic fluid from cancerous prostates than in the prostatic fluid from normal, inflamed, and hyperplastic prostates.
Eken et al., 2016 [32]	Patients with PCa exhibited significantly higher levels of manganese (Mn) and markedly lower levels of selenium (Se), while zinc (Zn) levels did not show substantial variance when compared to the control subjects.
Abdelmajid et al., 2022 [33]	The serum zinc level in cases exhibited a notable decrease compared to that of the controls.
Saleh et al., 2017 [34]	The tumorigenesis of prostate cancer seems to be linked with low selenium and zinc levels, as well as high manganese levels.
Nsonwu-Anyanwu et al., 2022 [35]	Decreased selenium levels were noted in the individuals with prostate cancer in comparison to the control group. The male participants with prostate cancer displayed reduced zinc levels in comparison to the control group. Elevated levels of lead were detected in the male participants with prostate cancer in comparison to the controls who were studied.
Ju-Kun et al., 2016 [46]	Elevated cadmium (Cd) exposure has been identified as a plausible risk factor for prostate cancer in occupational cohorts, although this association is not observed in nonoccupational groups. It is important to approach these findings with caution due to the substantial variability across studies.
Wu et al., 2021 [36]	Elevated levels of serum PSA were associated with higher blood Cd and blood Pb levels in men. No correlation was observed between elevated PSA and any of these heavy metal levels.
Tyagi et al., 2023 [5]	Elevated levels of cadmium (Cd) and arsenic were observed in individuals diagnosed with prostate cancer (PCa). The interplay between metal concentrations revealed a marked rise in Cd levels in smokers, indicating smoking status as a valuable indicator of heightened Cd levels and, consequently, an increased risk of PCa. The correlation between augmented metal concentrations and a higher incidence of PCa was evident. However, no significant variances were noted in the levels of lead (Pb) and nickel (Ni).
Lim et al., 2019 [37]	Arsenic (As), zinc (Zn), and manganese (Mn) exhibited substantial and favorable correlations with the risk of prostate cancer in the initial models. Favorable associations were observed between the serum concentrations of arsenic and zinc and the risk of prostate cancer when analyzed on the risk disparity scale using BKMR models.
Alegre-Martínez et al., 2022 [38]	Elevated levels of nickel have been observed in individuals with prostate cancer, with the concentration showing a correlation with the duration of exposure and age. Even low-level environmental exposure to nickel has been associated with reduced kidney function.
Pizent et al., 2022 [39]	The blood Hg level was notably higher in prostate cancer patients across both cohorts and the entire study population. Furthermore, patients from the Serbian cohort exhibited significantly elevated blood Cd levels and lower levels of As, Pb, and Ni, while cases from the Croatian cohort showed significantly higher serum Ni compared to controls. Even after matching the study groups by age, the statistical significance of the difference between patients and controls persisted for Hg and Ni in the Croatian cohort, and for Cr, Cd, Hg, and Pb in the Serbian cohort.
Qayyum and Shah, 2014 [40]	The levels of Pb, Cd, Ni, and Mn were markedly elevated in the blood and scalp hair of individuals with prostate cancer, in contrast to those in the control group. Conversely, the concentration of Zn in the patients was notably lower than that in the controls. Furthermore, the average levels of Zn were considerably higher in the scalp hair and nails of the control group; however, some elements (Cd, Ni, and Mn) were notably higher in the nails of patients compared to those of the control group.
Hood et al., 2023 [41]	Elevated levels of zinc were observed in the prostates of individuals with prostate cancer compared to those without the disease (*p* = 0.0116). Additionally, the prostate cancer cases demonstrated significantly higher levels of selenium (*p* = 0.0116) compared to their counterparts. Across the board, the average concentrations of metals were higher in individuals with prostate cancer than in those without. Furthermore, in a multivariate analysis, the metal profiles were found to be significantly distinct between the prostate cancer cases and the control group.
Keltie et al., 2022 [42]	The correlation between toenail total As and changes in toenail As speciation profiles is apparent. These data imply that toenails serve as a robust and valuable biomarker for studying the health effects of prolonged exposure to iAs.
Onyema-iloh et al., 2014 [43]	The average concentrations of selenium and zinc showed a statistically significant decrease (*p* < 0.05) in comparison to the control group.
Chang et al., 2018 [44]	Cadmium (Cd) and nickel (Ni) levels were notably elevated in individuals with BPH compared to the control group, while mercury (Hg) levels were most pronounced in patients with prostate cancer (PCa).
Pietrzak et al., 2024 [45]	The influence of combined Se and Zn levels on survival in prostate cancer patients is a topic of great significance. Despite the well-established impact of Zn, our data strongly suggest that optimizing both Se and Zn levels is more beneficial.

## Data Availability

All data sets associated with this publication are presented in this manuscript.
